# Cangfu daotan decoction for polycystic ovary syndrome: A protocol of systematic review and meta-analysis

**DOI:** 10.1097/MD.0000000000017321

**Published:** 2019-09-27

**Authors:** Hye-Rin Park, Jong-Hwan Kim, Donghun Lee, Hee-Geun Jo

**Affiliations:** a Chung-Yeon Korean Medicine Hospital, Gwangju; b Department of Herbal Pharmacology, College of Korean Medicine, Gachon University, Seongnam; c Chung-Yeon Medical Institute, Gwangju, Republic of Korea.

**Keywords:** cangfu daotan decoction, changbudodam-tang, polycystic ovary syndrome, randomized controlled trial, systematic review

## Abstract

**Backgrounds:**

Polycystic ovary syndrome (PCOS) is common endocrine disorder in women and can lead to serious social burdens associated with various reproductive and metabolic abnormalities. Existing therapy is controversial in its effectiveness including side effects. In traditional Korean Medicine, Cangfu Daotan Decoction (CDD), also known as Changbudodam-tang, is used for PCOS patients who are in the type of stagnation of phlegm and dampness. In this study, we aimed to evaluate the efficacy and safety of CDD for PCOS as alternative treatment.

**Methods:**

Two researchers will search the following databases from their inception to February 2019 for relevant randomized controlled trials (RCTs): The Cochrane Library, PubMed, EMBASE, Chinese National Knowledge Infrastructure Database (CNKI), and 5 Korean medical databases (Korean Studies Information Service System, KoreaMed, DBPIA, Oriental Medicine Advanced Searching Integrated System, and Research Information Service System). The primary outcome will be the scales that assessed drug efficacy including total response rate, sex hormone level (LH, FSH, Testosterone, LH/FSH ratio), BMI, ovulation rate, and pregnancy rate. Adverse events will be assessed as the secondary outcome. Study selection, data extraction, and assessment of risk of bias will be conducted by 2 researchers independently. Statistical analysis will be performed by using the Cochrane Review Manager (RevMan 5.3) software.

**Results and Conclusion:**

This review will provide the latest knowledge and evidence on the efficacy and safety of CDD for PCOS women through the analysis of various evaluation scales.

**Ethics and dissemination:**

This systematic review does not require ethical approval and will be published in a peer-reviewed journal.

**PROSPERO registration number:**

CRD42019134270.

## 1 Introduction

Polycystic ovary syndrome (PCOS) is a female disease characterized by hyperandrogenism, ovulatory dysfunction, and polycystic ovarian morphologic features.^[1]^ Although there are no internationally recognized standard diagnostic criteria and laboratory tests for PCOS diagnosis, Rotterdam consensus is the most widely used diagnostic guideline for PCOS, which is based on expert consensus-derived criteria.^[2]^ The Rotterdam criteria should include at least 2 of the following 3 characteristics: clinical or biochemical hyperandrogenism; oligo-anovulation; and polycystic ovaries, excluding other endocrinopathies (Cushing's syndrome, nonclassic adrenal hyperplasia, androgen-producing tumors, and drug-induced androgen excess).^[1]^ In the meta-analysis of 2016, proportions of PCOS prevalence were confirmed to be 8% to 13% based on the Rotterdam diagnostic criteria. Prevalence rates of hirsutism, hyperandrogenemia, polycystic ovaries, and oligo-anovulation were more than 10% in PCOS patients,^[3]^ and it can lead to a significant impact on women's health and quality of life. Also, PCOS patients have an increased risk of cardiovascular disease, 2.7 times the risk of endometrial cancer, and are associated with problems, such as pregnancy complications and emotional distress.^[4–7]^ Therefore, PCOS is associated with a number of severe reproductive and metabolic abnormalities, leading to a large social burden, thus early diagnosis and treatment are necessary.

Due to the complex pathology and causes of PCOS, clinicians and researchers are not fully aware of the underlying disease.^[8]^ Therefore, comprehensive treatments for PCOS have not been proposed yet, and since there are currently no drugs approved as PCOS indications, actual clinical treatment is dependent on individual patient's symptoms.^[9]^ Oral contraceptive pills (OCPs) are widely used to reduce menstrual irregularity and hyperandrogenism, and clomiphene citrate is used to induce ovulation in infertile patients.^[9]^ However, OCPs have been reported to increase the risk of venous thromboembolism, and there is a continuing debate over the lack of meaningful data supporting the effects of glucose metabolism.^[10,11]^ On the other hand, some experts have raised the issue that the long-term risk-benefit ratio is not clear, since the study period of many PCOS target chemical drugs is only a short 1 year or so.^[12]^ Because of these limitations of existing treatments, discussions about novel approach to PCOS patients are being actively made, and the interest in Traditional East Asian herbal medicine is increasing as one of these significant options.

In traditional East Asian Medicine, including traditional Korean Medicine, medicinal herb-based herbal formulas have been administered. Based on the multi-target mechanism, herbal formulas improve the complex symptoms of patients.^[13]^ Meanwhile, scientific evidence on herbal medicine for various gynecological diseases has been already accumulated, and recent studies have shown that many of the herbal medicines targeting PCOS can improve both reproductive and metabolic problems.^[14,15]^ In traditional Korean Medicine, there is no Korean medical term referring to PCOS, but it is considered as a category of irregular menstruation, infertility, obesity, etc. There are various herbal formulas for treating PCOS, and Cangfu Daotan decoction (CDD), also known as Changbudodam-tang, is one of the most common prescriptions for PCOS patients who are in the type of stagnation of phlegm and dampness.^[16]^ There have been numerous randomized controlled clinical trials (RCTs) using CDD in PCOS patients. Several systematic reviews and meta-analysis studies have been published in China, but they only included RCTs which assessed the efficacy of CDD used in combination with western medications.^[17,18]^ Therefore, this study will be conducted to evaluate the efficacy and safety of CDD for PCOS patients in various types of RCT studies including sole therapy as well as adjacent therapy.

## 2 Methods

### 2.1 Design and registration

A meta-analysis will be conducted to evaluate the efficacy and safety of CDD for PCOS as alternative treatment. This protocol has been registered on PROSPERO (ID: CRD42019134270). This protocol has been developed in accordance with the Preferred Reporting Items for Systematic Reviews and Meta-Analysis Protocol (PRISMA-P) statement guidelines.^[19]^

### 2.2 Study selection

#### 2.2.1 Study type

RCTs which used CDD or a combination of CDD and other conventional pharmacotherapy as treating PCOS will be eligible. Non-RCTs, quasi-RCTs, case reports, and animal studies will be excluded.

#### 2.2.2 Study object

Participants who are diagnosed with PCOS according to the Rotterdam criteria (2003) will be included. There will be no limitation on age, nationality, ethnicity, education or economic status and whether they were outpatients or inpatients. If the Rotterdam criteria is not mentioned in the trials, two researchers (HR, JH) will evaluate each trial's inclusion criteria and determine whether they are included or not through agreement.

#### 2.2.3 Intervention type

We will include trials using any type of CDD. CDD could be used alone or combined with other types pharmacotherapy. Including conventional western medication, if the control group received the same treatment as the intervention group, cointerventions were also allowed. We will include any type of control intervention including no treatment, placebo, conventional western medication, and other interventions except western drugs.

#### 2.2.4 Primary outcome

The primary outcome will include a scale that assessed drug efficacy. The included scales will be differences in total response rate, sex hormone level (LH, FSH, testosterone, LH/FSH ratio), BMI, ovulation rate, and pregnancy rate compared with those of the control group.

#### 2.2.5 Secondary outcome

The secondary outcome assessments will be adverse events.

### 2.3 Data sources and search strategy

The following databases will be searched from their inception to February 2019 for relevant studies: The Cochrane Library, PubMed, EMBASE, Chinese National Knowledge Infrastructure Database (CNKI), and 5 Korean medical databases (Korean Studies Information Service System, KoreaMed, DBPIA, Oriental Medicine Advanced Searching Integrated System, and Research Information Service System).

For a comprehensive search, the terms will be composed of several keywords including various symptoms of PCOS and various dosage forms of CDD, and they are as follows: polycystic ovarian syndrome, PCOS, amenorrhea, oligomenorrhea, hyperandrogenism, polycystic ovaries, ovulatory dysfunction, anovulation, hirsutism, infertility, and cangfudaotan decoction/granule/formula/tablet/pill/capsule. Table 1 shows that the search strategy for PubMed and modified search strategies will be applied to other databases.

**Table 1:**
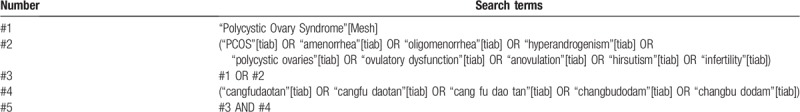
Search strategy for PubMed.

### 2.4 Study screening

**Figure 1:**
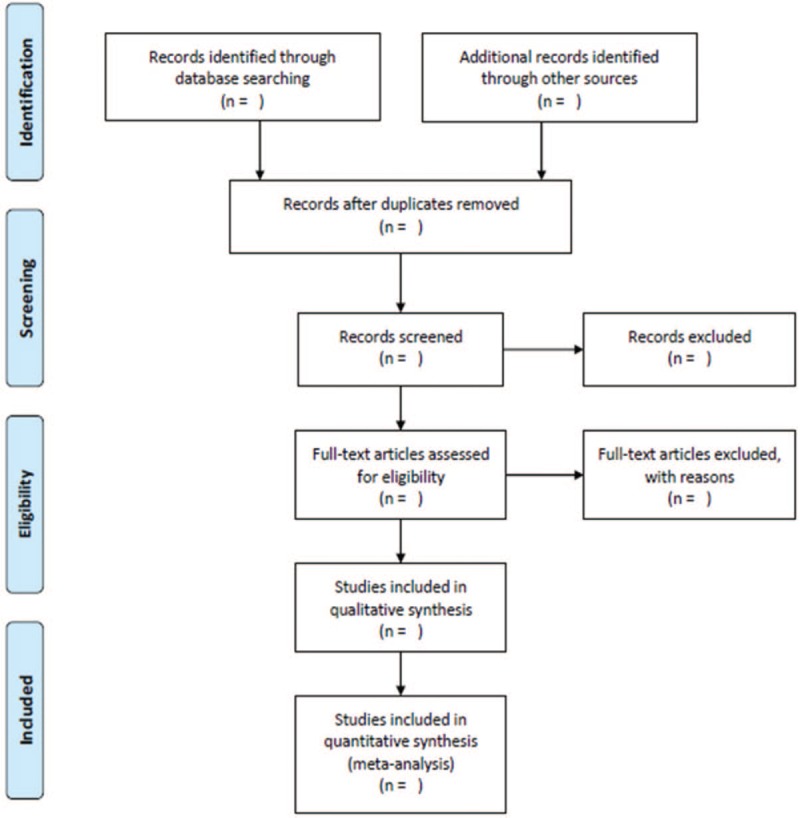
PRISMA flow diagram of study selection.

Two researchers will independently assess all relevant studies and select eligible articles that meet inclusion criteria by reviewing the titles and abstracts. The language will be limited to English, Korean or Chinese. The full-texts of articles will be examined for further evaluation. In case of discrepancy between review authors, an agreement will be made by the discussion with the corresponding author (HG). The procedure of study selection will be summarized by using a PRISMA flow diagram (Fig. 1).

### 2.5 Data analysis

#### 2.5.1 Data extraction

Two researchers will independently extract data from articles using a standardized data extraction form. The tabulated data will include the first authors, the country where the study was conducted, sample size, the inclusion/exclusion criteria of participants, details on the intervention and control groups, treatment duration, the outcomes, adverse events, and the composition of herbal medicine.

#### 2.5.2 Assessment of risk of bias

Two researchers will independently assess each included study for the risk of bias by using the Cochrane “risk of bias” tool for randomized controlled trials. The following risk of bias items will be assessed: random sequence generation, allocation concealment, blinding of participants, personnel and assessors, incomplete outcome data, selective reporting, and other sources of bias. Disagreements will be solved through the discussion with a third investigator when necessary.

#### 2.5.3 Assessment of heterogeneity

To evaluate heterogeneity between studies, *I*^2^ statistic and the chi-square test with a significance level of *P* < .1 will be used. If *I*^2^ statistic value is more than 50%, included studies are considered to have significant heterogeneity. In this case, we will conduct a subgroup analysis for further assessment of potential factors.

#### 2.5.4 Data synthesis

Statistical analysis will be performed by using the Cochrane Review Manager (RevMan 5.3) software when a meta-analysis is allowed. The extracted data were divided into dichotomous and continuous variables. We will pool the results using risk ratios (RR) with 95% confidence intervals (CI) for dichotomous variables, and mean differences (MD) with 95% CI for continuous variables. When *I*^2^ < 50%, the fixed-effect model will be selected and when *I*^2^ > 50%, the random-effect model will be selected. If the data quantitative synthesis is not possible, we will analysis the data qualitatively.

#### 2.5.5 Subgroup analysis

To identify substantial heterogeneity, we will conduct subgroup analysis according to the patient's characteristics, type of western medication, and the duration of herbal medicine treatment.

#### 2.5.6 Dealing with missing data

If there are insufficient or missing data in selected studies, we will contact the authors by e-mail and attempt to get missing data. When we cannot get that, we will only analyze the available data.

#### 2.5.7 Sensitivity analysis

We will conduct a sensitivity analysis to investigate the robustness of the study conclusions to the extent possible.

#### 2.5.8 Assessment of publication bias

If there are more than 10 articles for the meta-analysis, we will generate funnel plots to assess publication bias.

#### 2.5.9 Grading the quality of evidence

The GRADE system will be used for evaluating the quality of evidence in systematic reviews. The following 5 items will be assessed: risk of bias, consistency, directness, precision, publication bias, and additional points. The result of assessments will be graded into 4 levels: high, moderate, low or very low.

## 3 Discussion

PCOS is an endocrine disorder that can interfere with the quality of life in women and can be a high risk factor for type 2 diabetes mellitus and cardiovascular disease in overweight women.^[20–22]^

Various western medication treatments are used for PCOS therapy, but their use is controversial due to their side effects. For examples, common side effects of OCPs can include headache, mood changes, and weight gain.^[23]^ Clomiphene citrate, a selective estrogen-receptor modulator, can make side effects such as abdominal pain or discomfort, nausea, headache, hot flashes, and mood swings.^[24]^ As a result, complementary and alternative medicine treatments, such as acupuncture, moxibustion, and herbal medicine are attracting attention as an alternative treatment.

Among them, CDD is a herbal prescription widely used for PCOS patients who have been turned into a stagnation of phlegm and dampness type. Its original prescription is composed of Atractylodis Rhizoma, Cyperi Rhizoma, Aurantii Fructus Immaturus, Citri Unshius Pericarpium, Poria Sclerotium, Arisaematis Rhizoma Preparata cum Bovis Fel, and Glycyrrhizae Radix et Rhizoma. It is known to have the effect of removing the phlegm and dampness.^[16]^ There have been studies on the efficacy and safety of CDD, which has been used in combination with conventional medications for PCOS; but, to our knowledge, there is no systematic review or meta-analysis including RCT studies designed as CDD sole therapy (e.g., CDD versus western medicine).

Therefore, this review will be conducted to evaluate the efficacy and safety of CDD for PCOS women including various type of RCTs. However, it seems that there are some limitations due to the restriction of language, inconsistency of prescription composition, and difference in the dosage form.

## Author contributions

The study was conceptualized by HR. The protocol was drafted by HR and JH. The search strategy was developed by JH. DL revised the manuscript. HG supervised the study. All authors have approved the final manuscript.
